# The acceptability of high resolution anoscopy examination in patients attending a tertiary referral centre

**DOI:** 10.1186/s12885-018-4475-6

**Published:** 2018-05-11

**Authors:** Anke De-Masi, Esther Davis, Tamzin Cuming, Noreen Chindawi, Francesca Pesola, Carmelina Cappello, Susan Chambers, Julie Bowring, Adam N. Rosenthal, Peter Sasieni, Mayura Nathan

**Affiliations:** 1 Homerton Anal Neoplasia Service, Homerton University Hospital NHS Foundation Trust, London, E9 6SR UK; 2 KCL School of Cancer and Pharmaceutical Sciences, Guy’s Hospital, London, SE1 9RT UK; 3 University College London Hospitals NHS Foundation Trust, EGA Wing, Clinic 2, 235, Euston Road, London, NW1 2BU UK; 4 Wolfson Institute of Preventive Medicine, Queen Mary University of London, Charterhouse Square, London, EC1M 6BQ UK

**Keywords:** High resolution anoscopy, HRA, Patient experience, Quality of care, Anal high-grade squamous intraepithelial lesions, Anal HSIL

## Abstract

**Background:**

High resolution anoscopy (HRA) examination is regarded as the best method for the management of anal high grade squamous intraepithelial lesions to prevent anal squamous carcinoma. However, little is known about the acceptability of this procedure. This analysis looks at patient experience of HRA examination and ablative treatment under local anaesthetic.

**Methods:**

Patients took part in anonymised feedback of their experience immediately after their HRA examinations and/or treatments. A standard questionnaire was used that included assessment of pain and overall satisfaction scores as well as willingness to undergo future HRA examinations.

**Results:**

Four hundred four (89.4%) responses were received and all responses were analysed. The group consisted of 119 females (29.4%) and 261 males (64.6%) with median age of 45 years (IQR = 19) and 45 years (IQR = 21) respectively, and included 58 new cases, 53 treatment cases and 202 surveillance cases. 158 patients (39.1%) had at least one biopsy during their visits. The median pain score was 2 [Inter Quartile Range (IQR) 3] on a visual analogue scale of 0 to 10, where 0 indicated no pain / discomfort and 10 indicated severe pain. The median pain score was 2 (IQR 2) in men and 4 (IQR = 3) in women [Dunn’s Test = 4.3, *p* < 0.0001] and 3 (IQR 4.5) in treatment cases. Problematic pain defined as a pain score of ≥7 occurred more frequently in women (14%) than in men (6%), [Chi square test (chi^2^) = 5.6, *p* = 0.02]. Patient satisfaction with the care they received, measured on a scale of 0 (not happy) to 10 (very happy) found the median score to be 10 with 76% reporting a score of 10. Out of 360 responses, 98% of women and 99% of men said that they would be willing to have a future HRA examination.

**Conclusions:**

In this cohort, the overall pain scores were low and similar across appointment types. However, women had a higher pain score, including troublesome pain levels. Despite this, both women and men were equally satisfied with their care and were willing to have a future examination. The results of the analysis show that the procedure is acceptable to patient groups. A small number of women may need general anaesthesia for their examinations/treatment.

## Background

High risk human papillomavirus (HPV) infections are associated with lower anogenital tract cancers [1]. Rates of anal cancer and associated mortality rates have been predicted to increase over the next two decades in the United Kingdom [2]. High resolution anoscopy (HRA) consists of the examination of the anal canal and perianus using a magnifying device with a good light source (colposcope), after application of 5% acetic acid solution to highlight abnormalities that indicate anal neoplasia. HRA and directed biopsy is regarded as the definitive method for the detection of high grade squamous intraepithelial lesion (HSIL) in the anal canal and perianus [3] (collectively called anal, hereafter). Anal HSIL is considered as the precursor lesion for anal squamous cell carcinoma and the detection of anal HSIL will enable treatment or close monitoring to help with cancer prevention efforts [4]. Like colposcopy, HRA is a specialist skill that is learned over time [5]. Recently, the minimum standards for the practice of HRA have been published [6]. We do not yet have published data on the formal assessment of patient experience from those undergoing HRA examination and treatment that include both men and women. A previous study reported on patient experience of men in a screened population [7].

We conducted a study of patient experience at Homerton anal neoplasia service (HANS), a tertiary referral unit, run by a multidisciplinary team in the United Kingdom. Patients are referred from across the UK for anal and perianal HSIL diagnosis and treatment. Additionally, women with lower anogenital tract HSIL or suspected HSIL are referred to HANS for further management. The aim of the study was to establish if HRA, including biopsy and HRA-guided treatment, is acceptable as a procedure to a UK population of men and women.

## Methods

**Fig. 1:**
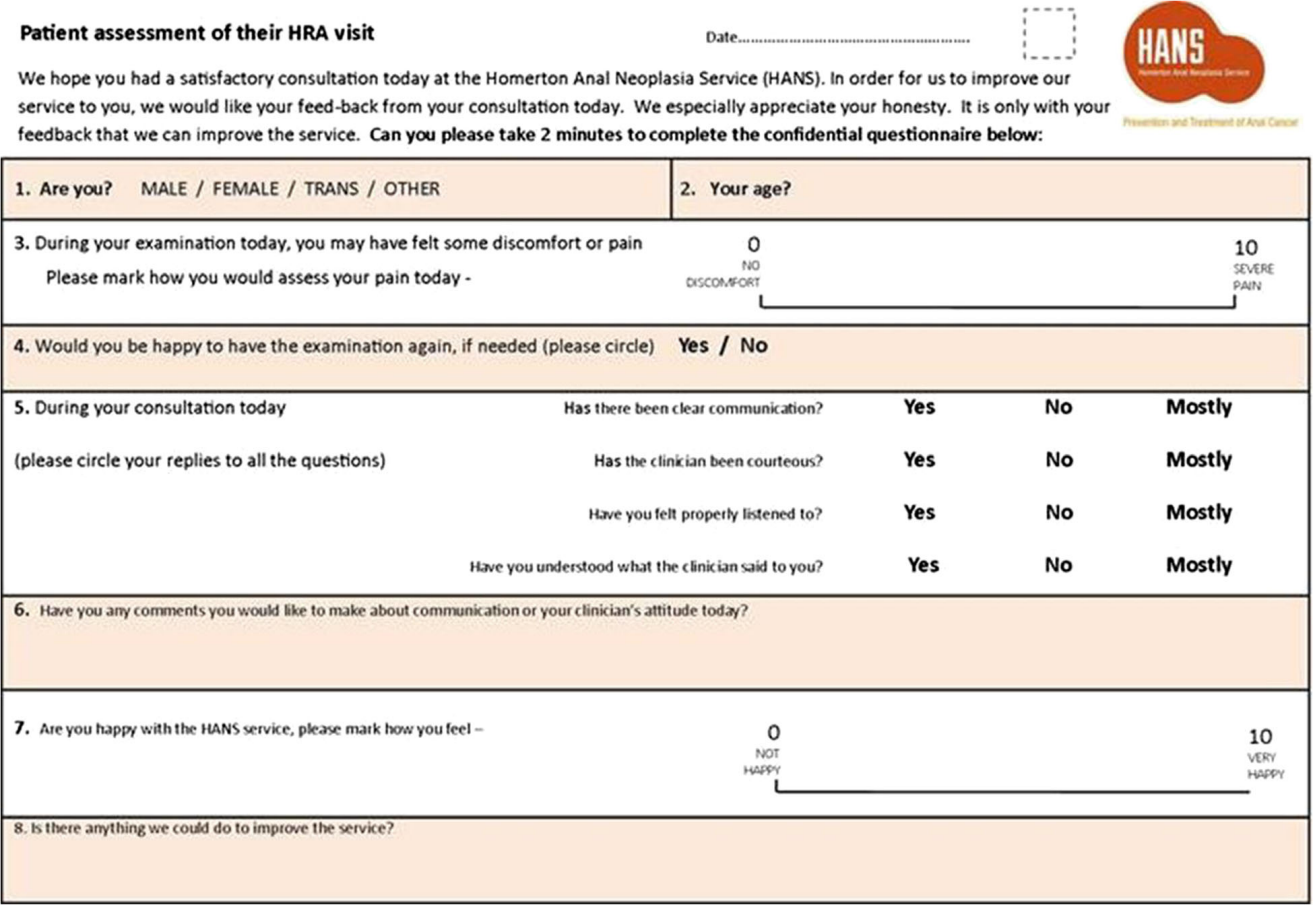
Patient feed-back form

During the period between October 2015 and August 2016, after obtaining institutional approval, patients attending HANS were asked to provide their feedback. Following verbal consent, patients were examined in the outpatient (office) setting in the dorsal lithotomy position with an adjustable bed. Patient assessments were made after 5% acetic acid applications to the zones to be inspected. Women had multizonal assessments that included examination of the cervix, vagina, vulva, perianus and the anal canal. Men had examination of the perianus and anal canal, unless they had genital symptoms or previous history of penile neoplasia, in which case genital examinations were additionally conducted. All biopsies were obtained after the administration of Citanest 3% with octapressin (injection, prilocaine hydrochloride 30 mg/mL, felypressin 0.03 unit/mL; Aston Pharma Trading ltd., 3016 Lake drive, Citywest Business campus, Dublin 24, Ireland) by injection, by using a Tischler biopsy forceps. Ferric subsulphate (Monsel’s solution) was then applied to the biopsy site for haemostasis. Patients attending for treatment with laser ablation applied EMLA™ cream 5% (contains lidocaine and prilocaine) to the treatment areas prior to arrival in the office. Treatment patients underwent HRA assessments to mark out the areas for treatment, then received local anaesthetic as above via submucosal or subcutaneous injections, and underwent treatment with CO_2_ laser ablation (for perianal disease) or diode laser ablation (for anal canal disease). Outpatient-based treatments were limited to disease involving no more than 2 quadrants (50%) of the circumference. Using a pre-formatted feedback form (Fig. 1), patient experience was collated. The questions used in the feedback form had face validity established with a small group of patients prior to the data collection. The feedback form also included a free text section inviting suggestions for improving the care they received. Duration of examination was not recorded. However, our clinic appointments are assigned in such a way that men have 30 min for their consultation and examination, while women have 1 hour for consultation and examination.

The forms were given immediately after the procedure to all consecutive patients during the study period by the nurse. The forms had a visual analogue scale of 0 to 10, where 0 indicated no pain or discomfort at all, and 10 indicated severe pain. The independent clinician and nurse scores of patient experience of pain were recorded. The nurse also made a note of the type of patient visit (assessment vs. follow-up vs. treatment) and the number of biopsies, if taken. The feedback form also included an overall satisfaction score; a visual analogue scale with 0 indicating ‘not happy’ and 10 indicating ‘very happy’. Patients were also asked about their willingness to undergo HRA examination in the future. Patients were asked to fill the form in privacy at the Unit’s reception and post them into a box kept at the reception. The forms were anonymous and had no patient identifiers other than age and sex. A retrospective analysis of the data was conducted after Institutional approval (Homerton Hospital Project number - 2377/2818).

## Results

During the period of October 2015 and August 2016, 452 patient attendances were recorded and 404 responses (89.4%) were received. Of the 404, 119 (29.4%) were females while 261 (64.6%) were males (24 missing gender). The median age was 45 years (IQR = 19) in females and 45 (IQR = 21) in males. There were 58 new cases, 53 treatment cases and 202 surveillance cases in the study population (data not entered in 101). 158 cases (39.1%) had at least one biopsy during their visits and amongst these patients 85 (54%) had 2 or more.

From 399 responses, the median pain score was 2 (IQR 3) on a pain scale of 0 to 10, where 0 indicated no pain or discomfort and 10 indicated severe pain. The median pain score amongst men was 2 (IQR 2) while in females was 4 (IQR 3) [Dunn’s Test = 4.3, *p* < 0.0001]. Overall, 57 patients (14.3%) reported a zero (0) pain score. In 157 cases who had biopsies during their visit the pain score was 3 (IQR 3). From 52 patients who had treatment under local anaesthesia, the pain score was 3 (IQR 4.5). Problematic pain, defined as a pain score of ≥7, occurred in a small number of cases (9%). More women (14%) reported problematic pain compared to men (6%) [chi^2^ = 5.6, *p* = 0.02]. Further analysis of problematic pain by visit type did not show significant differences (chi^2^ = 5.8, *p* = 0.06; Table 1). There was no correlation between the number of biopsies and the pain score (Spearman’s rho = 0.09, *p* = 0.14). The pain score values appeared consistent across patients and clinicians (Table 2).

**Table 1:** Problematic pain by visit type

Pain /discomfort levels	New cases (%)	Treatment (%)	Surveillance(%)	*p*-value
Low pain < 7	48 (84.2)	45 (86.5)	188 (93.5)	0.06
Problematic pain ≥7	9 (15.8)	7 (13.5)	13 (6.5)	
Total	57	52	201	

A visual analogue scale was used from 0 to 10, where 0 meant no discomfort or pain, while a score of 10 indicate severe pain. The median pain score from 399 responses was 2 (IQR 3). Problematic pain score is defined as a score of ≥7. Problematic pain (32/399) identified in 6% of men and 14% of women (chi^2^ 5.6, *p* = 0.02)

**Table 2:** a and b comparison of pain scores by patients and clinicians

a				
Patient – pain score	Nurse assistant – pain score			Total
	0–3	4–6	≥ 7	
0–3	176	47	7	230
4–6	37	27	6	70
≥7	10	13	2	25
Total	223	87	15	325
b				
Patient – pain score	HRA clinician - pain score			Total
	0–3	4–6	≥ 7	
0–3	103	45	6	154
4–6	21	23	4	48
≥7	2	12	4	18
Total	126	80	14	220

Clinicians independently assessed the pain after the procedure and made a note before handing the form for the patient to fill-in in private. Pain scale consisted of a numerical visual analogue scale where 0 indicates no pain or discomfort felt by the patient, while 10 indicates severe pain. There is an association between nurse and patient pain scores (Fisher’s exact test *p* < 0.001). Similarly there is an association between clinician’s and patient’s pain scores (Fisher’s exact test *p* < 0.001)

Feed-back on overall satisfaction with care received was obtained on a scale of 0 to 10, where 0 meant ‘not happy’ with the service while a score of 10 indicated ‘very happy’ with the care. The median score from 368 responses (91.1%) was 10, with 76% reporting a score of 10. Although 24% reported a score of less than 10, only 4% scored 7 or less on happiness with their care. There were no differences noted between men and women. A further patient response, that related to ‘the willingness to a future HRA examination’, was collected. 44 patients (10.9%) did not answer this question. Of those that answered, 99% were willing to re-attend. 4 patients were not (Table 3). No differences were observed between men and women regarding their willingness (99% of men and 98% of women) for a future HRA examination.

**Table 3:** Willingness for future HRA examinations

Patient category	Yes	No / unsure	No response
After HRA	88.1% (356 / 404)	1% (4 / 404)	10.9% (44 / 404)
After treatment	90.6% (48 / 53)	0	9.4% (5 / 53)
After biopsy (≥1 bx)	87.3% (138 / 158)	1.3% (2 / 158)	11.4% (18 / 158)

Total population = 404. 158 cases (39.1%) had at least one biopsy during their visits and amongst these patients 85 (54%) had 2 or more

## Discussion

This study reports the immediate feedback on the procedure of HRA by patients. This procedure is for the detection of anal cancer precursors, and in those at high risk, needs to be carried out regularly in order to maintain surveillance. From the patients who filled out the relevant section of the survey, the procedure including taking of biopsies and treatment does appear to be highly acceptable to them. Current management of anal HSIL in many areas of the UK involve multiple biopsies under general anaesthetic, usually without the high-resolution element to allow targeting of biopsies. HRA involves not only directed biopsies, hence fewer in number, but is carried out as a no/ local anaesthetic procedure.

Prospective evaluation of a service has the advantage of ensuring that data collection can be adequately planned, and pain assessment is contemporaneous and is more likely to be complete. By employing this method, we had an excellent response rate to this evaluation.

There is an increasing trend in healthcare evaluation to ensure that patients’ views and opinions are taken into account http://www.healthknowledge.org.uk/public-health-textbook/research-methods/1c-health-care-evaluation-health-care-assessment/study-design-assessing-effectiveness (accessed on 5/11/2017). This enables the analysis of health care provision from the patients’ rather than healthcare providers’ perspective.

The team at Homerton anal neoplasia service (HANS) consists of a number of HRA practitioners and this study reflects the overall performance of the whole team at HANS. It may be possible for us to bench-mark patient initiated scores of pain and overall satisfaction of care for our service, for comparison with other services, as well as individual practitioners in the future. Patients at high-risk of anal carcinoma include HIV-positive men and women [8], those on immune-suppressants such as renal transplant recipients [9] or patients with systemic lupus erythematosus [10], and the experience of HRA examination may vary in different patient groups. Although the HRA practice standards have been published and will help guide HRA practice, an important element of assessing performance will be to utilise patient experience.

Rates of overall questionnaire answering were high but not all patients completed all the questions. This was the price of anonymity which we felt was important, in order to encourage truthful answers. HRA involves intimate examination and biopsy under local anaesthetic.

Treatment with laser ablation is one form of ablative treatment for anal HSIL. In a randomised controlled study comparing 3 different treatments for anal neoplasia, pain was assessed as a side effect to treatment [11]. This study did not distinguish between pain felt during the procedure and pain that occurred during the recovery period. The pain assessment was retrospective and not contemporaneous in nature. This study indicates that pain during the procedure for small office-based ablative procedures under local anaesthetic is acceptable.

In our study, pain scores were essentially similar between the new cases, treatment cases and those attending for surveillance. Although ‘problematic pain’ defined as a pain score of ≥7 was uncommon in this cohort of patients, a relatively larger number of women reported ‘problematic pain’. This may relate to the fact that women have multizonal assessment which includes examination of the cervix, vagina and vulvar regions at the same visit as high resolution anoscopy. Previous anecdotal observations suggest that the duration of examination may determine the level of pain or discomfort experienced by the patient. The guidelines for international practice standards recognises the duration of HRA examination to be important [6]. In the light of our study’s findings, going forward, we will have a lower threshold for offering women multizonal HRA assessment under general anaesthesia.

We compared nurse and physician assessment of pain with patients’ own pain (Table 2a and b). There were 2 patients out of 220 with clinician scores of 0–3, where the patient recorded a pain score of 7 or more. The opposite case, where a high score was assigned by the clinician, but the patient rated this at 0–3 occurred in 6 out of 220. Nursing evaluation missed severe pain in 10 / 325 and overestimated the pain in 7 / 325. Overall, correlation was fairly accurate with assessment by nurses and clinicians, hence abandoning the office procedure if pain is experienced, and rebooking it with sedation or general anaesthetic is a possible solution for that small percentage who found the procedure painful.

In our cohort of patients, women were equally satisfied with the care they received as men and almost all men and women were willing to return for a future examination. In a study looking at the psychological impact of being screened for anal cancer in HIV-positive men who have sex with men, patients were more likely to have higher negative impact scores immediately after being screened, compared to at other time points such as pre-screen and post-results [12]. This supports the timing of patient acceptability feedback that we carried out. Further, patient’s recollection of their pain experience seems to rely on the peak intensity of the pain during the procedure and on the intensity of the pain recorded during the last 3 min of the procedure, when measured for colonoscopy and lithotripsy [13]. This may explain the occasions when recollected patient pain score did not correlate with clinician-awarded pain score.

HRA examination is thought to be the ideal method for the diagnosis of high grade anal neoplasia, through directed biopsies [14]. It enables a reduction in anal cancer progression rates when used for diagnosis, treatment and surveillance of anal HSIL [15]. Preliminary data indicate that HRA may help to reduce local disease failure of T1–3 anal cancer cases (TNM classification), when used for surveillance after treatment [16]. It is noteworthy that anal HSIL often occur in association with anal squamous carcinoma, and is believed to be the precursor to anal carcinoma [4]. HRA surveillance enables detection and adequate treatment of anal HSIL.

This study has several limitations. The National Health Service in the UK advocates routine collection of patient feed-back on services. We obtained patient feed-back over a 10-month period, from patients seeing different members of staff. Due to the anonymous nature of the survey, we could not stratify the results according to the clinician who saw the patient. A small number of patients (~ 5%) may have attended twice and filled out two separate forms. This may have ‘amplified’ feed-back results. Our patient cohort consisted of those who had HRA only, those who had one or more biopsies, and those who received treatment. The patients undergoing treatment and surveillance were by definition groups who had already attended at least once previously and thus may bias the results in favour of patients willing to return for a further examination. However, there were no significant differences between these three groups in terms of willingness to return.

No sample size calculations were conducted but we found a difference in pain scores between men and women; further studies and replication of the findings are necessary. The overall response rate was high it was not 100%, and not all patients answered all questions. This may have reflected a failure of trust in the anonymization process, leading to a refusal to fill out any deemed to be a critical or negative response. Both these factors could bias the results in favour of those who experienced less pain/were more willing to return. Finally, the feedback audit was conducted in a tertiary referral unit, and thus the results may not be generalizable to other populations.

Our results are encouraging in that due to low pain scores and a high proportion of patients being willing to return for further visits, we feel that HRA can be supported as an outpatient procedure, including for small volume ablative treatments. Access to general anaesthetic or sedation may be required for patients, particularly women, who may experience pain during examination and for larger volume ablative treatments.

## Conclusion

High resolution anoscopy (HRA) in the outpatient setting including biopsy and ablative treatment under local anaesthetic is well-tolerated in men and women in a tertiary referral centre in the UK. Treatment and biopsies did not impact on the acceptability and pain scores of the procedure. A small number of people may require general anaesthetic or sedation in order to undergo the procedure in comfort. We propose that units currently carrying out non-high resolution anoscopy with mapping biopsies under GA consider training in HRA and transferring surveillance of anal intraepithelial neoplasia (AIN) to this less invasive outpatient-based modality.

## Abbreviations

Chi ^2^: Chi square test
CO2 laser: Carbon dioxide laser
HANS: Homerton anal neoplasia service
HPV: Human papillomavirus
HRA: High resolution anoscopy
HSIL: High-grade squamous intraepithelial lesion
IQR: Interquartile range
SD: Standard deviation
